# Optimizing fluconazole-embedded transfersomal gel for enhanced antifungal activity and compatibility studies

**DOI:** 10.3389/fphar.2024.1353791

**Published:** 2024-03-28

**Authors:** Zhiqiang Cheng, Ujjwala Kandekar, Xiaoshi Ma, Vishal Bhabad, Ashlesha Pandit, Liming Liu, Jiping Luo, Neha Munot, Trushal Chorage, Abhinandan Patil, Sandip Patil, Liang Tao

**Affiliations:** ^1^ Zhongshan School of Medicine, Sun Yat-Sen University, Guangzhou, China; ^2^ Department of Pathology, The Second Affiliated Hospital of Southern University of Science and Technology, Shenzhen, Guangdong, China; ^3^ Department of Pharmaceutics, JSPM’s Rajarshi Shahu College of Pharmacy and Research, Pune, Maharashtra, India; ^4^ Department of Pharmaceutics, Rajmata Jijau Shikashan Prasarak Mandal College of Pharmacy, Pune, Maharashtra, India; ^5^ Department of Pharmacognosy, JSPM’s Charak College of Pharmacy and Research, Pune, Maharashtra, India; ^6^ Department of Pharmaceutics, D. Y. Patil Education Society, Kolhapur, Maharashtra, India; ^7^ Department of Haematology and Oncology, Shenzhen Children’s Hospital, Shenzhen, China

**Keywords:** transfersomes, fluconazole, topical application, transfersomal gel, *ex-vivo* studies

## Abstract

Fungal infections are of major concern all over the globe, and fluconazole is the most prevalently used drug to treat it. The goal of this research work was to formulate a fluconazole-embedded transfersomal gel for the treatment of fungal infections. A compatibility study between fluconazole and soya lecithin was performed by differential scanning calorimetry (DSC). Transfersomes were formulated by a thin-film hydration technique using soya lecithin and Span 80. A central composite design was adopted to prepare different formulations. Soya lecithin and Span 80 were chosen as independent variables, and the effect of these variables was studied on *in vitro* drug diffusion. Formulations were evaluated for entrapment efficiency and *in vitro* drug diffusion. The results of *in vitro* drug diffusion were analyzed using the analysis of variance (ANOVA) test. Optimized formulation was prepared based on the overlay plot and evaluated by scanning electron microscopy, DSC, vesicle size, polydispersity index (PDI), zeta potential, and *in vitro* drug diffusion studies. An optimized formulation was loaded into xanthan gum gel base and evaluated for pH, viscosity, *in vitro* and *ex vivo* drug diffusion, and antifungal activity. DSC studies revealed compatibility between fluconazole and soya lecithin. Entrapment efficiency and *in vitro* drug diffusion of various formulations ranged between 89.92% ± 0.20% to 97.28% ± 0.42% and 64% ± 1.56% to 85% ± 2.05%, respectively. A positive correlation was observed between *in vitro* drug diffusion and Span 80; conversely, a negative correlation was noted with soya lecithin. Entrapment efficiency, particle size, zeta potential, PDI, and drug diffusion of optimized formulation were 95.0% ± 2.2%, 397 ± 2 nm, −38 ± 5 mV, 0.43%, and 81 % ± 2%, respectively. SEM images showed well-distributed spherical-shaped transfersomes. *In vitro*, *ex vivo* drug diffusion and antifungal studies were conclusive of better diffusion and enhanced antifungal potential fluconazole in transfersomal formulation.

## Introduction

Skin serves as a major barrier of protection, integrated all over the body and exposed to the surrounding environment. It is more likely to be affected by environmental allergens and infections (Smith et al., 2017). Various bacterial, viral, and fungal species are prone to attack the dermal layer and disturb its function. Topical fungal infections are more common, and they are difficult to eradicate. Billions of individuals all over the world are estimated to have hair, nail, and skin fungal infections. The severity of infections ranged from asymptomatic mild to potentially life-threatening infections and exhibited an impact on the quality of life. These outputs urge to focus on the development of antifungal agents and various formulations thereof. The antifungal drug fluconazole is a third-generation triazole drug and is widely used for the treatment of systemic and superficial fungal infections (Lee et al., 2021). It acts by inhibiting fungal cytochrome P-450 enzyme, thereby restricting the production of ergosterol (integral part of the fungal membrane) (Guengerich et al., 2022). The inhibitory action is more profound in fungal species than that in mammalian cells; this characteristic contributes to the safety profile of triazole in humans. Irrespective of the broad spectrum of activity and good safety profile, it has limited solubility, requires more doses orally, and has several negative impacts mainly, headache, diarrhea, stomach pain, liver, and kidney damage etc. Topical dosage forms are available in the market; however, they still face challenges due to limited solubility and skin permeation (Gupta et al., 2010). Topical drug delivery is an effective means to treat several dermatologic disorders (Kaur and Guleri, 2013). Despite this, the tight keratin layer limits the efficacy of formulations. This issue can be resolved by the development of new strategies for topical delivery. Vesicular system transfersomes are made up of an inner aqueous compartment encircled by a lipid bilayer and edge activator (Walve et al., 2011). The lipid bilayer constitutes a membrane, while edge activators contribute to flexibility and permeability. These are deformable, elastic, and squash themselves as intact vesicles with no loss via small pores of the dermal layer (Sivannarayana et al., 2012). They exhibit the ability to entrap hydrophilic and lipophilic moieties and possess a more flexible structure than liposomes, owing to the presence of edge activators (Bhasin and Londhe, 2018). The rationale of current research work is to formulate transfersomes of fluconazole to enhance its solubility and ultimately antifungal activity.

## Materials and methods

Fluconazole was sourced from Aarti Drugs Ltd, Tarapur, India, while soya lecithin and Span 80 were procured from Research Lab Fine Chem Industries, Mumbai, India. All other chemicals used were of analytical grade.

### Drug–excipient interaction study by differential scanning calorimetry

Thermal properties were estimated by differential scanning calorimetry (DSC). Pure drugs, soya lecithin, and physical mixture were subjected to DSC studies. In a sample pan, 1–2 mg of the sample was placed, and an empty pan was served as the reference. The sample was run over a range of 25°C–300°C with a heating rate of 100°C/min (Castelli et al., 2005).

### Preparation of transfersomes

Different formulations were prepared using the thin-film hydration method in two steps, as reported by Opatha et al. (2020) . Fluconazole, soya lecithin, and Span 80 were precisely weighed and dissolved in 30 mL of a (2:1 v/v) mixture of chloroform and methanol. After the complete dissolution of soya lecithin and drug in the organic mixture, solvents were evaporated using a rotary evaporator (EQUITRON Rotary evaporator-Evator, India) by maintaining the temperature of water bath at 51°C with 100 rpm until the thin film was formed at a wall of a round bottom flask. Later, 25 mL of phosphate buffer (pH 5.5) was incorporated in a flask to hydrate the dried lipid film for 3 h. Finally, transfersomal dispersion was left for 1 h, which resulted in the formation of a multilamellar vesicle. Formulations were further sonicated using a probe sonicator (Digital Ultra, India) to obtain unilamellar vesicles with uniform particle size. Prepared transfersomes were stored at 4°C until further use. Trials were taken to screen the concentration of soya lecithin and Span 80, and different formulations were prepared by central composite design (Design Expert^®^ Software, Version 11.0, Stat-Ease, United States). Independent variables soya lecithin (X₁) and Span 80 (X₂) were selected in a range of 200 mg–400 mg and 1 mL and 1.5 mL, respectively. *In vitro* drug diffusion of fluconazole was selected as the dependent variable (Table 1).

**TABLE 1:** Formulation of transfersomes by central composite design.

Formulation	Drug (mg)	Soya lecithin (mg)	Span 80 (mL)
B1	50	400	1
B2	50	300	1.25
B3	50	158.57	1.25
B4	50	200	1
B5	50	400	1.25
B6	50	441.42	1.25
B7	50	300	0.896
B8	50	300	1.603
B9	50	400	1.5
B10	50	200	1.5

### Characterization of transfersomes

#### Determination of the entrapment efficiency (%)

The indirect method was used for the determination of entrapment efficiency (Pandit et al., 2023). Transfersomal dispersion was placed in centrifugation tubes and centrifuged at 8,000 rpm (C-24 plus, Remi Elektrotechnik Ltd., India) at 4°C for 2 h, followed by decantation of a clear supernatant solution. A clear decanted solution (1 mL) was diluted with methanol up to 10 mL and analyzed at 260 nm using a UV-visible spectrophotometer (UV1800, Shimadzu Corporation, Japan). Entrapment efficiency was calculated as entrapment efficiency (% EE) = (total amount of drug added–the amount of free drug in the supernatant/total amount of drug added) X 100.

#### *In vitro* drug diffusion study of transfersomes

Diffusion of fluconazole from transfersomes was evaluated using a Franz diffusion cell apparatus (DBK Instruments, Mumbai, India). Transfersomal dispersion (corresponding to 10 mg of fluconazole) was accurately weighed and transferred on a cellophane membrane (150 LA 401, HiMedia, Mumbai, India). A membrane was introduced between the receptor and donor compartment. The receptor compartment was filled with phosphate buffer at pH 5.5 as the diffusion medium. The medium was stirred with a magnetic stirrer and maintained at a temperature of 37°C ± 0.5°C. A sample (1 mL) was removed periodically at 30 min intervals, and the same volume of the diffusion medium was introduced to the cell to maintain the sink condition. The sample was further diluted by pH 5.5 phosphate buffer to 10 mL volume, and the absorbance was measured at 260 nm.

### Selection of the optimized formulation

The optimized formulation was meticulously chosen based on the impact of soya lecithin (X1) and Span 80 (X2), identified as independent variables, on drug diffusion (dependent variable Y). To assess this effect, an analysis of variance (ANOVA) test was used. The ANOVA test is a statistical method utilized to analyze variations in the means of different formulations and determine the significance of these variations. In our study, various formulations of transfersomes were prepared, and the values of *in vitro* diffusion were systematically analyzed using Design Expert Software. This software allows for a comprehensive examination of the responses to the required attributes, aiding in the determination of the probability of achieving the desired levels for the independent variables. The desirability function played a crucial role in our methodology, enabling the analysis of responses for the essential attributes and facilitating the prediction of the likelihood of reaching the desired level for the independent variables. Subsequently, an overlay plot, utilizing a desirability value of unity, was constructed. This plot served as a valuable tool in the identification and selection of the optimized formulation, ensuring that it met the desired criteria for enhanced drug diffusion.

### Characterization of the optimized formulation

#### Scanning electron microscopy

Dispersion of transfersomes was sonicated for 5 min before testing. Dispersion (1 mL of) was diluted with the freshly prepared phosphate buffer pH 5.5. The drop of dispersion was applied over carbon taps on the aluminum stub and air-dried. The dried sample was subjected to SEM studies by the model (SEM Quanta 200–EDX system, ICON laboratories, Mumbai, India) (Sarwar et al., 2018).

#### Measurement of the vesicle size, polydispersity index, and zeta potential

Particle size, polydispersity index (PDI), and zeta potential were measured using Zetasizer (Horiba-SZ100, Japan) (Duangjit et al., 2013). Transfersomal dispersion was sonicated using a probe sonicator for 5 min before testing. Furthermore, 0.5 mL of transfersomal dispersion was diluted with phosphate buffer at pH 5.5 and exposed to dynamic light scattering. Each test was carried out in triplicate (Sohail et al., 2018).

#### Differential scanning calorimetry study

For the DSC thermogram of the optimized formulation, we followed a method analogous to the one described for the drug–excipient compatibility study. In this case, the formulation was positioned in a sample pan, and an empty pan served as the reference. It is noteworthy that, unlike the drug–excipient compatibility study where pure excipients were used, the DSC study for the optimized formulation involved the use of the actual formulation in the sample pan. This nuanced approach was undertaken to provide a more accurate representation of the thermal properties of the formulated product. In response to concerns about the specific lipid and edge activator chosen for this study, we will incorporate a brief but comprehensive explanation in the article to justify our selection, ensuring that this aspect is adequately addressed.

### Preparation of the transfersomal gel (T-gel) and plain gel (P-gel)

An optimized batch of transfersomes was selected for gel preparation. Xanthan gum (1 gm) was dispersed in distilled water (100 mL) and soaked overnight at room temperature. An accurate amount of transfersomes equivalent to 100 mg of the drug was added to gel base by continuous stirring using a magnetic stirrer (Bio Technics, India) for 20 min until a homogenous gel was formed. The prepared transfersomal gel was stored at 4°C for further examination. The gel loaded with fluconazole was also formulated using the same method. For both formulations, 0.1% sodium benzoate and glycerin were added as a preservative and humectant, respectively.

### Evaluation of the gel

#### Measurement of pH

pH of the gel was measured using a digital pH meter (LMPH-9, MAA Scientific Instrument Lab, India) (Gupta and Singhvi, 2012). In a 10 mL beaker, 5 gm of gel was placed, and the pH was measured at room temperature in triplicate (Yadav et al., 2019).

#### Viscosity determination

The transfersomal gel and marketed gel (M, gel, Flumet, Leeford Healthcare Ltd) were tested for the measurement of viscosity using a using a viscometer (LVDV, Brookfield, USA). In a beaker, 10 gm of gel was placed, and the viscosity of the gel was measured using spindle 64 at 30, 60, and 100 rpm (Gul et al., 2022).

#### *In vitro* drug diffusion from gel formulation

*In vitro* drug diffusion from the transfersomal and the marketed gel was carried out using a Franz diffusion cell apparatus (Waqas et al., 2022). The transfersomal and marketed gel dose corresponding to 10 mg of fluconazole was weighed precisely and transferred to the cellophane membrane. The cellophane membrane was sandwiched between the receptor and donor compartment. The rest of the evaluation was performed similarly, as described under *in vitro* drug diffusion of transfersomal formulations.

#### *Ex vivo* skin diffusion and drug skin retention studies

An *ex vivo* skin permeation study was accomplished through the goat skin (hair removed from the dorsal side) using a Franz diffusion cell. pH 5.5 phosphate buffer was used as a receptor medium, maintained at 35°C ± 0.5°C. Transfersomal gel (T-gel), gel loaded with pure fluconazole (P gel), and marketed gel (M gel) were mounted on goat skin separately, further introduced between a receiver and donor compartment. At regular intervals, 1 mL of the sample was withdrawn and replaced with a fresh medium to maintain the sink for up to 7 h. Samples were analyzed using a UV-visible spectrophotometer at 260 nm. After completion of the test, the goat skin was cautiously detached, and the formulation attached to the skin was scraped off. The skin was cut into small pieces and immersed in a 10 mL mixture of methanol and phosphate buffer solution with pH 5.5 (Kumar, 2015). The mixture was stirred for 30 min, and the extract was analyzed using a UV-visible spectrophotometer at 260 nm to determine the drug retained in the skin.

#### Texture analysis of the transfersomal gel

Different properties of the gel formulation such as tackiness, firmness, stringiness, and work of adhesion were tested using a Brookfield texture analyzer CT3-100 (Brookfield Engineering Labs, Inc., United States). The test was fixed at the compression mode by means of probe TA3/100 and TA-BT-KIT fixture, and trigger load was kept at 5.0 g. T-gel (without any air pockets into the gel sample) was filled into the female probe. A tapering 35-mm male probe at an angle of 45°C was enforced down into individual specimens at a rate of 0.5 mm/s to a 10-mm depth. From the resulting time plot, cohesiveness (work essential to distort the hydrogel in the downward movement of the probe), hardness (strength obligatory to achieve a specified deformation), and adhesiveness (energy essential to surpass attractive forces among the sample and probe) were read carefully. Spreadability was calculated from the energy required to deform the sample (Pandit et al., 2023).

#### Antifungal activity of the transfersomal gel

The antifungal activity of the transfersomal gel (T-gel) and marketed gel (M-gel) was checked against *Candida albicans* using the agar well diffusion method (Rajan and Vasudevan, 2012). Precisely, 2.1 gm of Mueller–Hinton agar (MHA) was incorporated into a 250-mL a conical flask composed of distilled water (100 mL). It was thoroughly heated to obtain a clear solution. The resultant solution was sterilized for 15 min at 15 lb pressure at 121°C using an autoclave. The sterile agar solution was added to the MHA solution. The resulting medium was poured into a sterilized petri plate and allowed to solidify. Strains of *C. albicans* were streaked on a plate. Wells were created in sterile media with a sterile borer approximately at a depth of 6 mm. Finally, samples were transferred into wells and incubated at 37°C for 48 h (Choudhary et al., 2021). The zone of inhibition around the well was measured.

## Results and discussion

Fluconazole is an imidazole derivative and is employed to treat fungal infections. Oral fluconazole has many side effects such as headache, diarrhea, nausea, and stomach pain; to avoid such side effects, it was loaded in transfersomes and further incorporated into the gel for topical delivery. These are mainly composed of edge activators and phospholipids. Phospholipids show vesicle-forming potential and are used to create lipid bilayers. Edge activators are bilayer-softening materials that make vesicles more flexible and enhance skin permeability via self-optimizing deformability. The edge activator also increases the solubility and entrapment efficiency of a hydrophobic drug (Duangjit et al., 2011).

### Drug–excipient interaction studies by DSC

**FIGURE 1:**
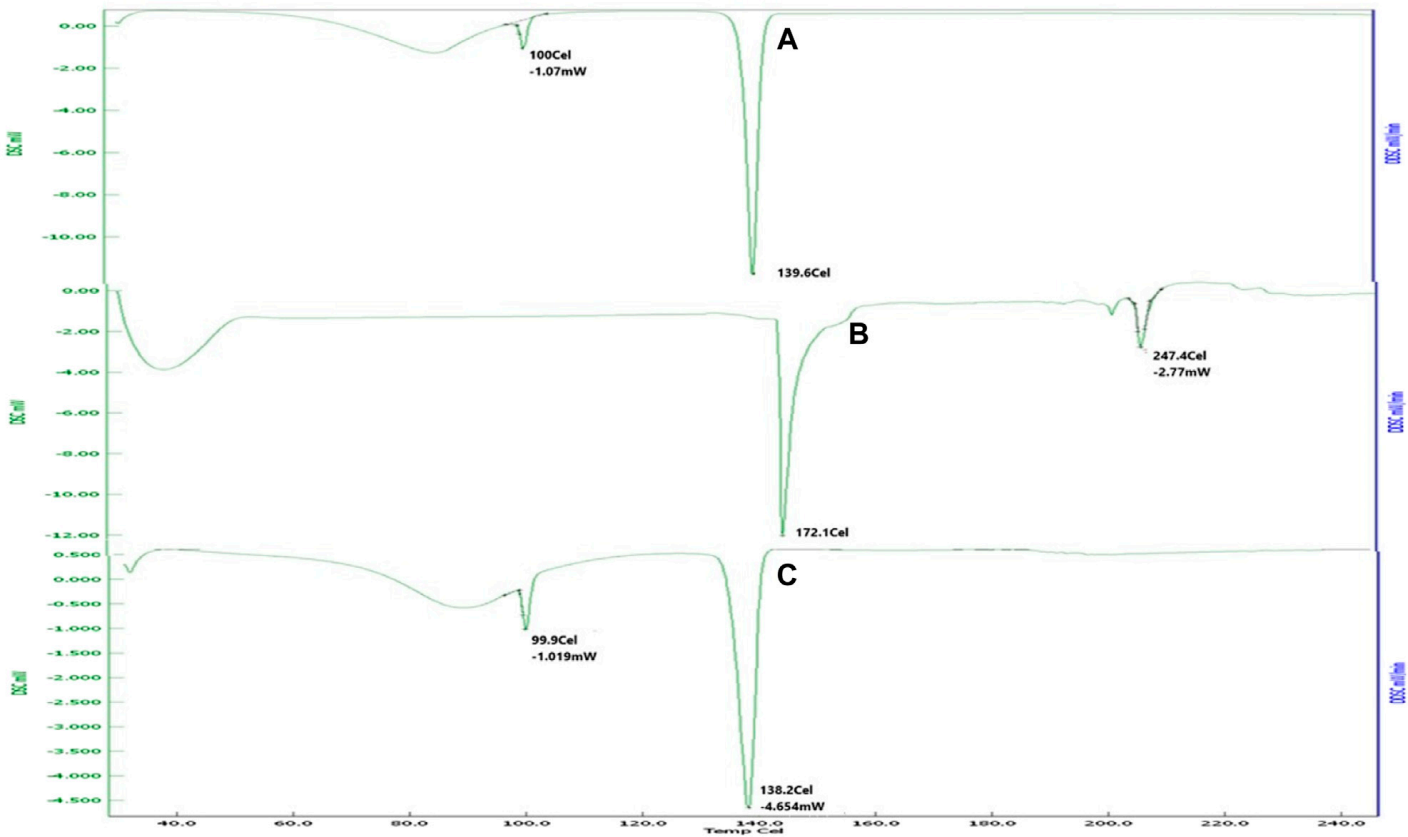
DSC thermogram of **(A)** fluconazole showed sharp melting at 139.6°C. **(B)** Soya lecithin indicated sharp melting at 172.1°C and 247.4°C. **(C)** Physical mixture revealing melting of the drug at 138°C, suggestive of intactness of the drug in the presence of soya lecithin.

DSC is mainly used as a thermal analytical tool to provide information about the drug–excipient incompatibility. The DSC curve for pure fluconazole (Figure 1) showed a sharp endothermic peak at 139.6°C, which was ascribed to the melting point of the drug (El-Housiny et al., 2022). Soya lecithin showed a sharp melting point at 172.1°C and 247.4°C. Soya lecithin is hygroscopic and composed of bound water; the peak observed at 247.4°C may be attributed to the isotropic liquid phase due to the release of bound water upon heating. These results were found in agreement with studies carried out by Yusuf et al. (2014) where a peak at 251°C was associated with the release of bound water (Fang et al., 2001). A physical mixture of fluconazole and soya lecithin (1:1) shows a peak endothermic peak at 138°C that is suggestive of the intactness of the drug in the presence of soya lecithin.

### Preparation of transfersomes

For preparation of transfersomes, soya lecithin and Span 80 were selected as the phospholipid and edge activator, respectively (Li et al., 2015). Soya lecithin was selected as a phospholipid because it can entrap lipophilic drugs, and it offers good encapsulation properties with a controlled release of drugs from transfersomes (Pandey et al., 2014). Span 80 was selected as an edge activator, owing to biocompatibility and a small vesicular size suitable for skin penetration (Lei et al., 2013). The thin-film hydration method was adopted to prepare transfersomes as the vesicle formed was stable and had good entrapment efficiency. The impact of temperature and time of hydration on the entrapment efficiency and vesicle size was studied by varying the temperature in the range of 45°C–55°C and hydration time 1–3 h. Slower evaporation of the solvent from the film occurred at 45°C–52°C, which allowed sufficient time for the drug to entrap. Increased temperature causes rapid evaporation of the film due to over-boiling that results in cracking, less drug entrapment, and deformed vesicles. Hence, a temperature of 51°C was selected as the optimum temperature. At lower hydration time, deformed vesicles were formed while enhanced hydration time up to 3 h resulted in well-discrete vesicles with a uniform size. Therefore, the films were hydrated for 3 h. Similar experimental conditions were reported by Varia et al. (2022).

### Characterization of transfersomes

#### Determination of the entrapment efficiency

**FIGURE 2:**
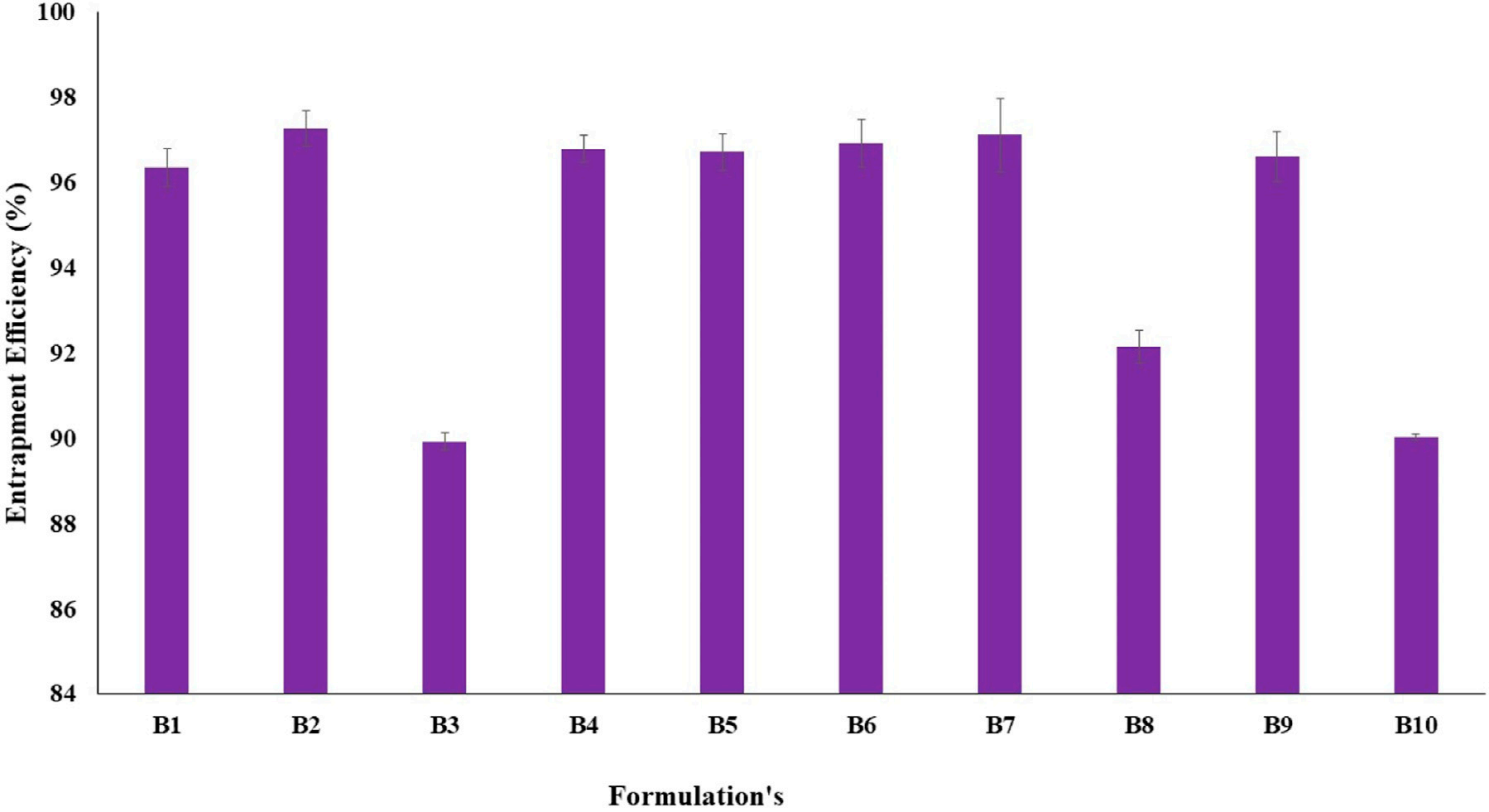
Drug entrapment efficiency of various formulations of transfersomes representing good entrapment of fluconazole in vesicles.

Fluconazole is soluble in methanol, whereas soya lecithin found in the outer layer of transfersomes is insoluble. Soya lecithin was precipitated out, and fluconazole was dissolved after the addition of methanol, which ultimately correlated with the quantity of free drug and consequently drug entrapped into vesicles. The entrapment efficiency of transfersomes ranged between 95.03% ± 0.08% and 97.28% ± 0.42% (Figure 2). Fluconazole is a moderately lipophilic drug; therefore, it has a greater affinity toward the lipid matrix. The amount of both the lipid and surfactant had a major influence on entrapping drugs in the vesicular system (Durán-Lobato et al., 2013). Soya lecithin contributed to the formation of more rigid vesicles to avoid the leaking of drugs from vesicles. Conversely, the enhanced concentration of Span 80 reduced drug entrapment, owing to the solubilization of lecithin and the generation of a more porous membrane, causing an enhanced leakage of the drug. Initial trials were conducted to optimize the amount of soya lecithin and Span 80. Different formulations were selected to achieve maximum entrapment.

#### *In vitro* diffusion of transfersomes loaded with fluconazole

**FIGURE 3:**
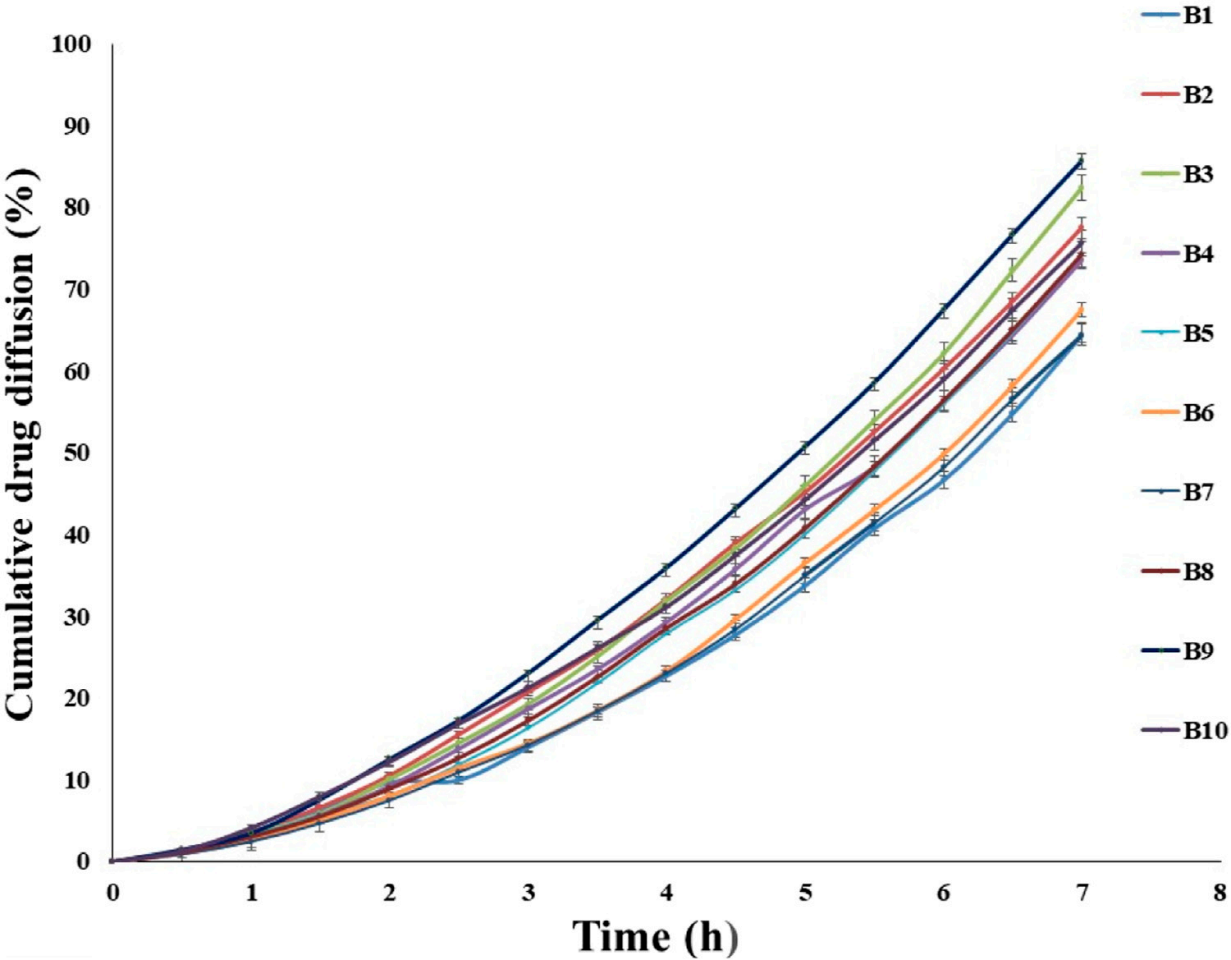
*In vitro* diffusion of fluconazole from various transfersomal formulations for 7 h depicting the impact of soya lecithin and Span 80 on diffusion of fluconazole.

**FIGURE 4:**
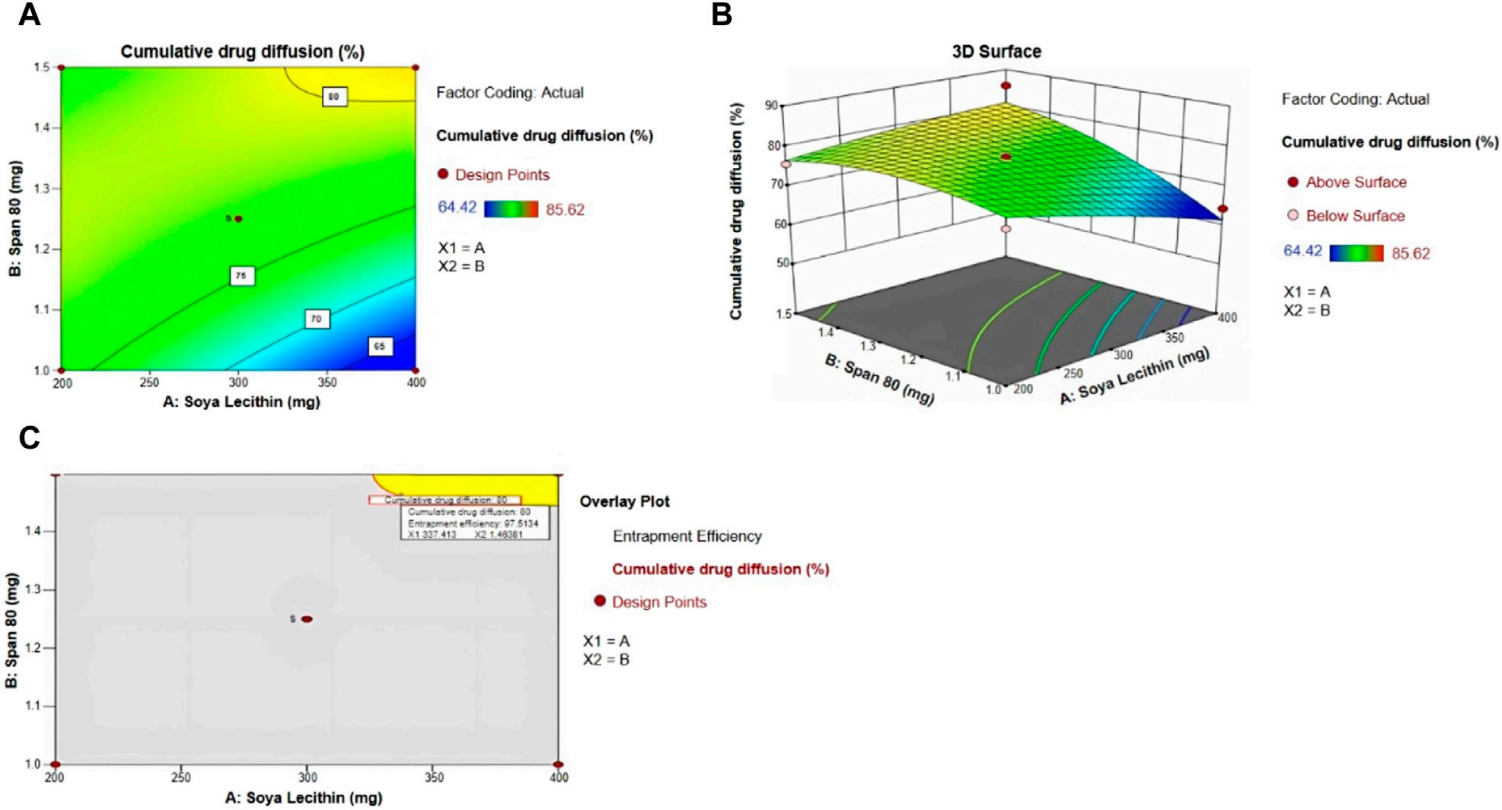
**(A)** Contour plot, **(B)** 3D surface plot, **(C)** and overlay plot, indicative of factors affecting drug diffusion and the optimum concentration of soya lecithin and Span 80 for desired drug diffusion.

The diffusion of the drug from the transfersomes was performed over 7 h at a temperature of 37°C in a phosphate buffer with pH 5.5 (Figure 3). The diffusion of the drug from transfersomes ranged between 64% and 85%. The difference in the diffusion pattern from transfersomes is attributed to the difference in the concentration of Span 80 and soya lecithin. The concentration of the surfactant had a direct correlation, while phospholipids had an inverse correlation for drug diffusion. The reason for this might be enhanced solubilization of fluconazole by Span 80, and drug retardation was endorsed by the formation of a compact bilayer structure by soya lecithin. Span 80 contains lipophilic tails with long and unsaturated (C18) moieties, which leads to a permeable vesicle membrane (Balata et al., 2020). Formulations B9 and B10 showed a higher drug diffusion of 85.62% ± 0.5% and 75.66% ± 0.3%, respectively, within 7 h, as they were composed of a higher amount of the surfactant. Conversely, slower diffusion was noticed in formulations B2, B3, B5, and B6, with diffusion rates of 77.45% ± 0.52%, 82.44% ± 0.41%, 74.28% ± 0.3%, and 67.48% ± 0.51%, respectively, within 7 h, as they were composed of a higher amount of soya lecithin (El-Gizawy et al., 2020). The diffusion data are indicative of the impact of soya lecithin and Span 80; hence, these variables were designated as independent variables, and drug diffusion was chosen as the dependent variable. Drug diffusion from different formulations was analyzed using the ANOVA test, and graphs were obtained using Design Expert Software. The contour plot and 3D surface (Figures 4A, B) of drug diffusion revealed that the enhanced concentration of Span 80 was responsible for higher drug diffusion, while the increased amount of soya lecithin retarded the diffusion of fluconazole. The ANOVA equation also supports these results, where a negative value associated with soya lecithin showed drug retardation and a positive coefficient of Span 80, indicative of faster drug diffusion. The *p*-value 0.0096 (<0.05) revealed the significance of the test.

$$Y=77.45‐2.53*A+5.01*B+4.76*\text{AB}‐0.7150*A^{2}‐2.99*B^{2}.$$

By taking into account soya lecithin and Span 80, the concentration overlay plot was obtained, as shown in Figure 4C. The overlay plot helped select an optimized formulation. The concentration of soya lecithin and Span 80 in the optimized formulation was chosen to achieve a prolonged release with maximum entrapment efficiency. The yellow region in the overlay plot showed a concentration of 337 mg of soya lecithin and 1.46 mL of Span 80 to achieve the desired drug diffusion of 80% within 7 h. The prepared formulation was evaluated further. The entrapment efficiency of optimized transfersome formulations was 95.0% ± 2.2%.

#### Scanning electron microscopy

**FIGURE 5:**
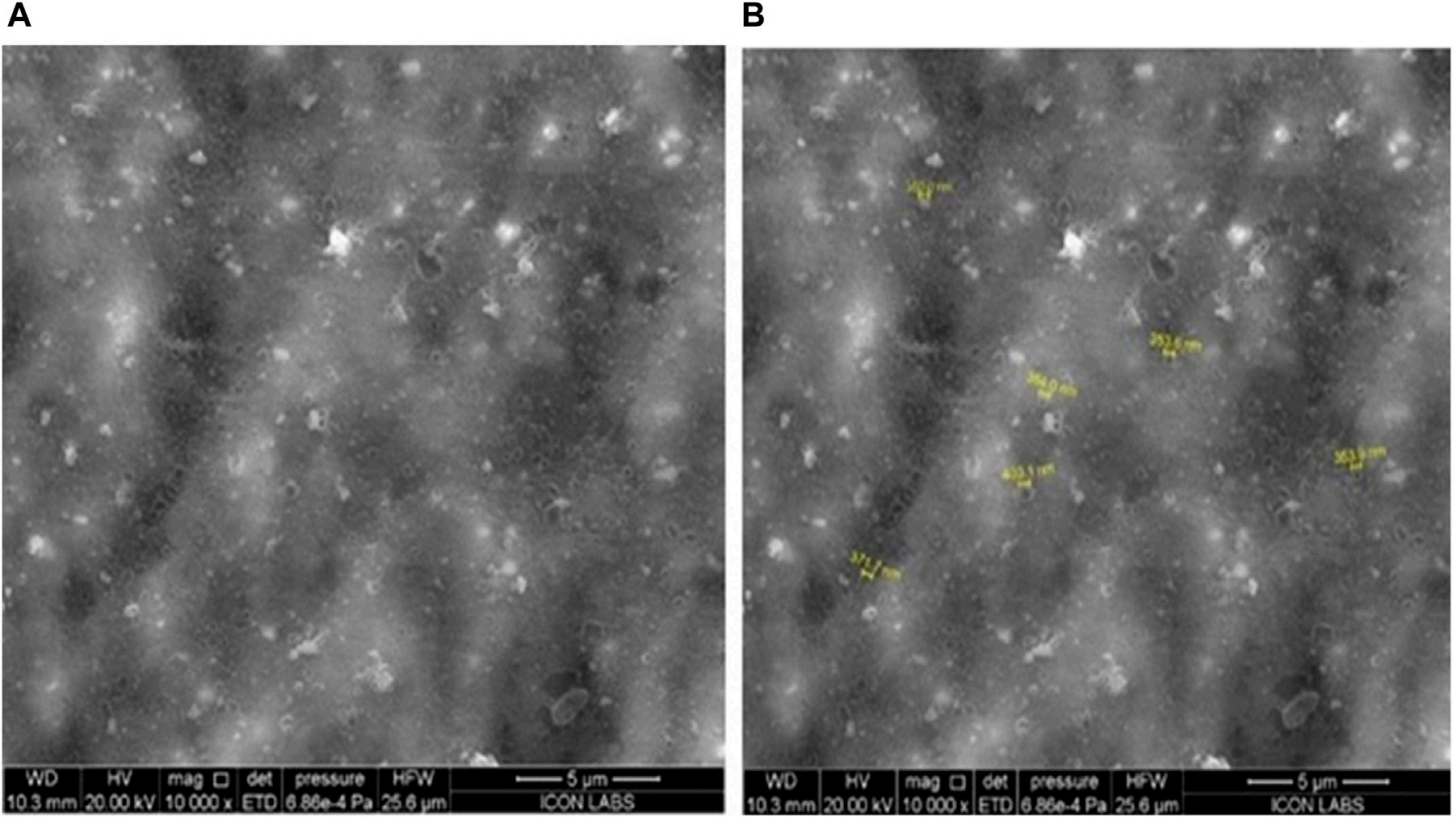
Characterization of optimized transfersomes: a scanning electron microscopy study on uniform distribution and spherical morphology for effective vesicular drug delivery.

The morphology of optimized transfersome vesicles was studied by SEM. SEM images were confirmative of the good distribution of transfersomes with a size range of 350–403 nm (Figure 5). Spherical morphology of transfersomes confirmed the vesicular drug delivery system.

#### Measurement of the vesicle size, polydispersity index, and zeta potential

**FIGURE 6:**
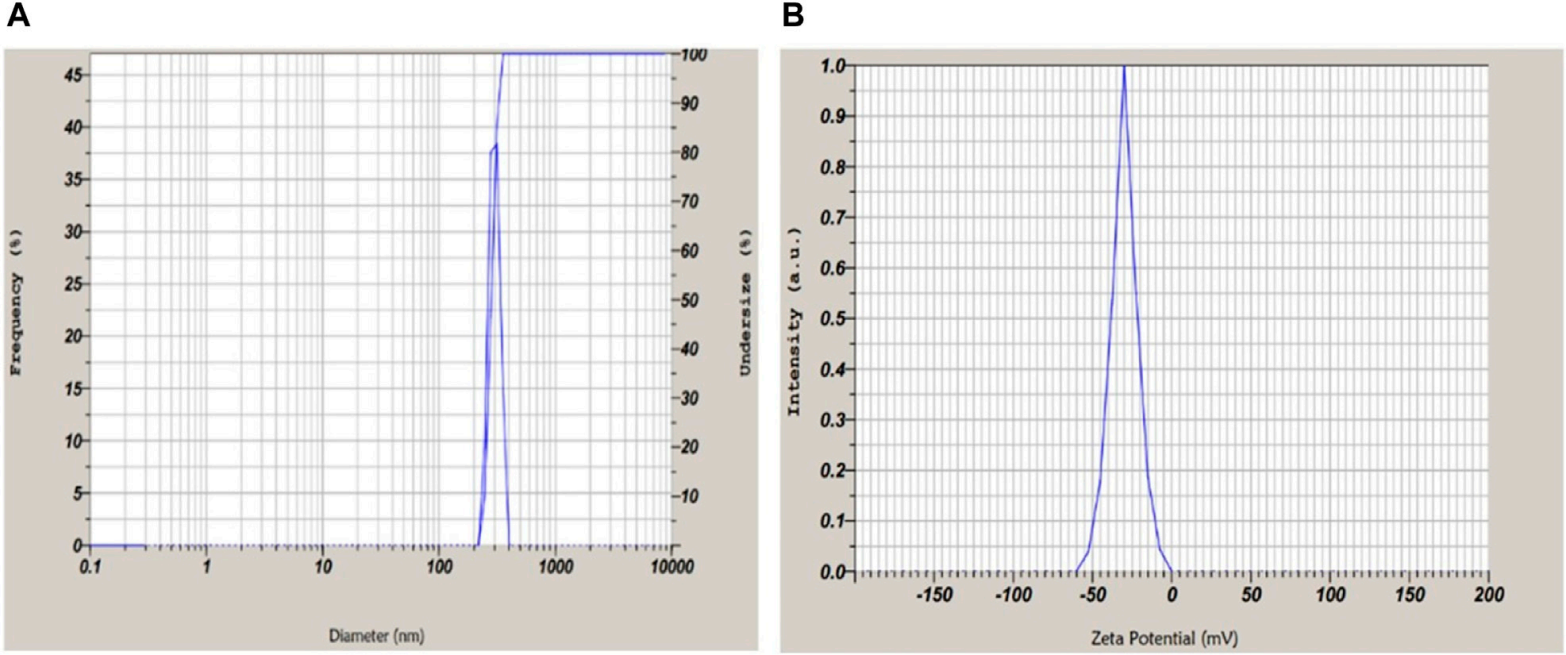
**(A)** Particle size and PDI suggestive of mono-dispersed microscopic particles. **(B)** Zeta potential of optimized formulation −38 ± 5 mV revealing stable and dispersed particles.

The average particle size of the optimized formulation of transfersomes was 397 ± 2 nm (Figure 6A), which is the optimum size of a vesicle for a topical drug delivery system. The PDI of the colloidal system indicates the distribution of particles in the system. Well-distributed particles in the system are indicative of a stable colloidal system. The PDI value below 0.7 denotes the monodispersity of dispersion (Massadeh et al., 2020). The PDI value for optimized transfersome formulation was 0.43, which indicated the system was moderately monodispersed. To study surface charges of transfersomes, zeta potential was determined as it is of crucial importance to stabilize the dispersion. Zeta potential values ranged between +30 and −30 mV, indicative of a high probability of flocculation of vesicles. The value for zeta potential was −38 ± 5 mV (Figure 6B, which was supportive of a better stability of transfersomes. The type of surfactant with their concentration affects the value of zeta potential (Tawfeek et al., 2020). The negative charge indicated more stability and enhanced penetration potential of formulation through the skin.

#### DSC study

**FIGURE 7:**
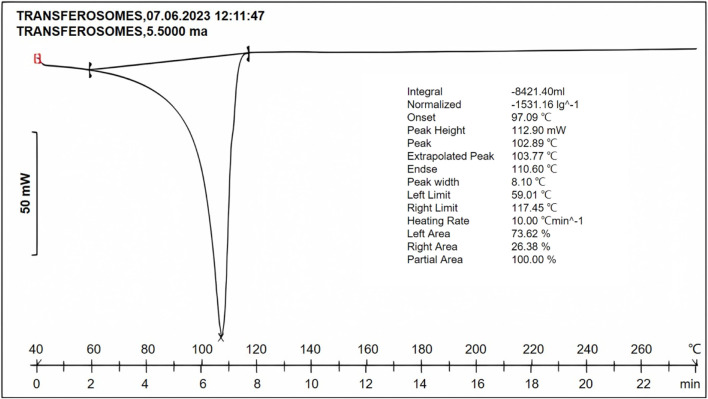
DSC thermogram of the optimized formulation indicating the peak at 102.89°C, suggestive of reduction in the melting point of the drug, owing to solubilization in the lipid phase and surfactant.

Transfersome formulation exhibited a peak at 102.89°C (Figure 7), and pure fluconazole exhibited a sharp peak at 139.6°C. The reduction in the peak was confirmative of the solubilization of the drug into the lipid phase.

#### *In vitro* drug diffusion study of the optimized formulation

**FIGURE 8:**
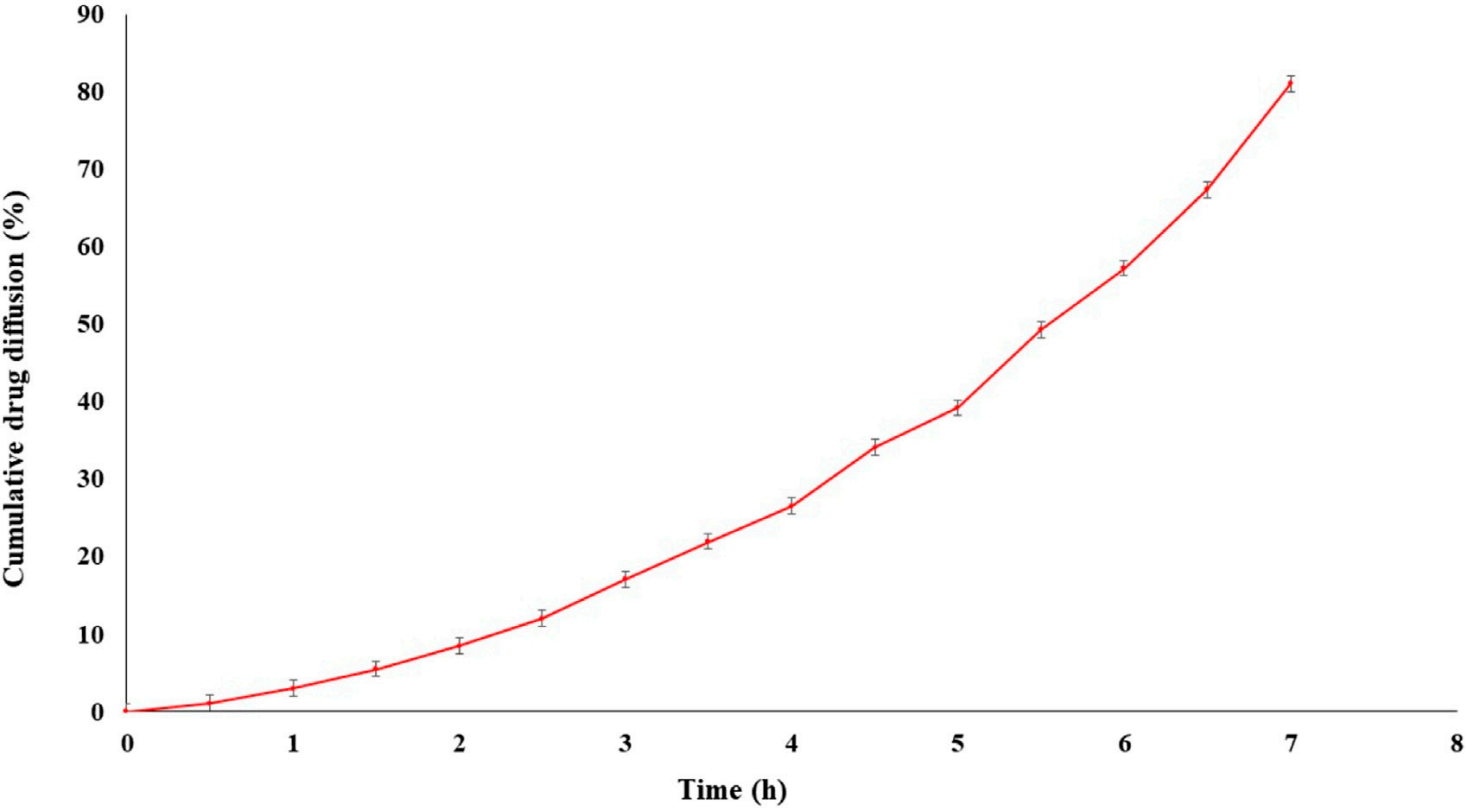
*In vitro* diffusion of transfersomes loaded for the optimized formulation showed drug release up to 7 h.

The drug diffusion from the optimized formulation exhibited a remarkable result, with a diffusion rate of 81.05% ± 0.23% within 7 h (Figure 8). The sustained drug diffusion, as discussed earlier, can be attributed to the presence of soya lecithin.

### Preparation of the transfersomal gel and pure fluconazole-loaded gel

The optimized formulation and pure fluconazole were incorporated in a gel base containing 1% xanthan gum as a gelling agent separately and evaluated.

### Evaluation of the transfersomal gel

#### Measurement of pH

The pH of the transfersomal gel and the plain gel was recorded thrice using a digital pH meter. The results ranged between 5.5 ± 0.23 and 5.6 ± 0.54 for the transfersomal gel and 5.1 ± 0.24 to 5.2 ± 0.41 for plain gel formulation. This pH range falls within the acceptable range of 4–7 for transdermal application (Pathan et al., 2018), indicating the suitability of the gel formulation for application on the infected skin.

#### Viscosity determination

The viscosity of gel formulations is an important parameter for ease of removal from the container and application on the skin, reflecting the consistency of the formulation (Shinde et al., 2012). The viscosity of transfersomal and marketed gel formulation was measured at different rpm values such as 30, 60, and 100 at room temperature with a Brookfield viscometer using spindle no. 64. The viscosity decreased with an increase in the rate of shear, displaying non-Newtonian flow characteristics (shear-thinning). This behavior is advantageous due to its low flow resistance when applied under high-shear conditions (Bousmina, 1999). Determinations were done in triplicate (Table 2).

**TABLE 2:** Viscosity of the transfersomal gel and marketed gel.

Rotation speed (rpm)	Viscosity of the transfersomal gel (Cp)	Viscosity of the marketed gel (Cp)
30	22,400 ± 1.64	11,133 ± 4.61
60	5,655 ± 3.45	6,219 ± 3.31
100	3,803 ± 5.30	4,389 ± 2.24

#### *In vitro* drug diffusion form gel formulations

**FIGURE 9:**
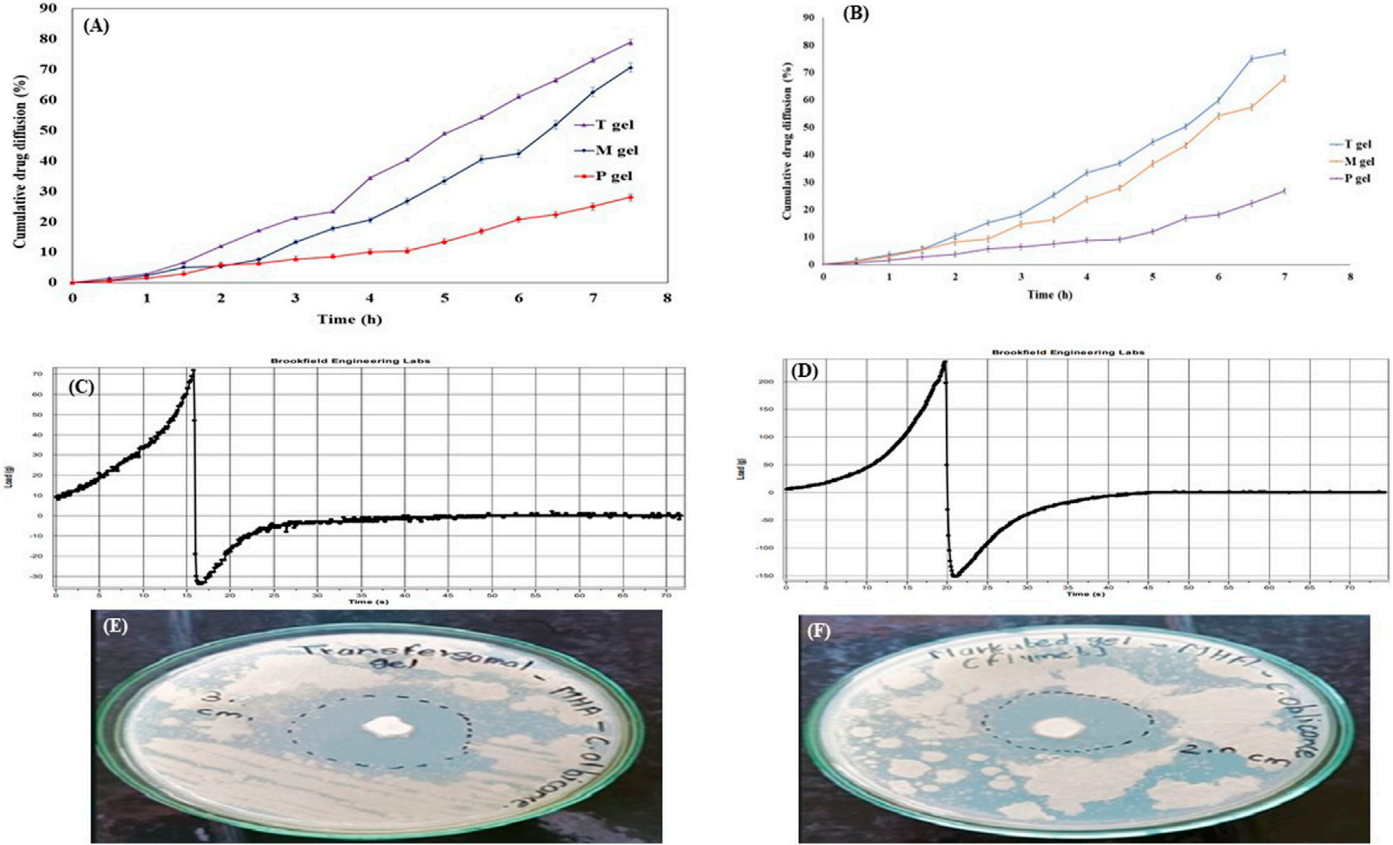
Evaluation parameters for the transfersomal gel. **(A)** *In vitro* drug diffusion from gel formulations for 7 h. **(B)** *Ex vivo* drug diffusion from gel formulations for 7 h. **(C)** Texture analysis profile of the transfersomal gel. **(D)** Texture analysis profile of the marketed gel showed better spreadability of the transfersomal gel. **(E)** Zone of inhibition for the transfersomal gel was 30 ± 0.9 mm. **(F)** Zone of inhibition for the marketed gel was 20 ± 1.2 mm, indicating more antimicrobial potential of the transfersomal gel.

The diffusion rates of the drug from the T-gel, M-gel, and P-gel were 78.85% ± 0.9%, 70.64% ± 1.5%, and 28.10% ± 1.2%, respectively, within 7 h (Figure 9A). The highest drug diffusion was from the T-gel, followed by the M-gel and P-gel. The reason for this might be attributed to the faster diffusion of transfersomes into skin layers. Transfersomes can change their membrane flexibility, contributing to the easy passage of drugs through the skin layer spontaneously. This ability is known as the self-optimizing deformability (Barani et al., 2021). As transfersomes are extremely deformable, these can easily cross the skin layer with very small pores (Yang and Merlin, 2020). A gel containing pure fluconazole showed minimum diffusion, owing to its highly lipophilic nature.

#### *Ex vivo* studies

T-gel formulation significantly enhanced the diffusion of fluconazole across the goat membrane, as compared to the M-gel and P-gel. Drug diffusion was 77.32% ± 0.17%, 67.76% ± 0.38%, and 26.77% ± 0.23%, respectively, within 7 h (Figure 9B). The quantity of the drug reserved in the skin for these formulations was 21.86% ± 0.31%, 17.70% ± 0.45%, and 9.18% ± 0.61%, respectively.

#### Texture analysis of the transfersomal gel

The texture analysis of the transfersomal gel focused on firmness, tackiness, and work of adhesion, which collectively contribute to cohesion, stickiness, and spreadability of semisolids. The results, presented as firmness (gm) and tackiness (gm) in comparison to the marketed gel (Figures 9C–F), indicate superior spreadability of the transfersomal gel over the marketed gel (Kolimi et al., 2022). These values were suggestive of the better spreadability of the transfersomal gel as compared to the marketed gel.

#### Antifungal activity of the transfersomal gel

The antifungal activity of the transfersomal gel was evaluated against a commercial gel using the agar well diffusion method on *C. albicans*. The antifungal activity was measured in terms of the zone of inhibition (Elmoslemany et al., 2012). Due to its widespread impact on humans, *C. albicans* was chosen as an infecting fungus. The zone of inhibition of the transfersomal gel was 30 ± 0.9 mm, and for the marketed gel, it was 20 ± 1.2 mm (Figures 9E, F), which demonstrates the good antifungal activity of the transfersomal gel against *C. albicans*.

## Conclusion

Our research successfully formulated and optimized fluconazole-loaded transfersomes incorporated into a xanthan gum gel base, demonstrating the enhanced solubility and efficacy of fluconazole. Through a series of *in vitro*, *ex vivo*, and antifungal studies, the transfersomal gel exhibited suitability for topical application, emerging as a promising carrier for fluconazole. This study addresses a critical gap in drug delivery for fungal infections, presenting transfersomes as an innovative approach to overcome solubility challenges and improve therapeutic outcomes. The developed transfersomal gel stands as a superior alternative for enhanced fluconazole delivery, highlighting its potential for future clinical applications. Further research avenues should explore strategies to improve the formulation stability and prevent oxidative degradation, potentially through the use of biodegradable polymers for coating transfersomes.

## Data Availability

The original contributions presented in the study are included in the article/Supplementary Material; further inquiries can be directed to the corresponding authors.
